# Characteristics of Hemodynamic Disorders in Patients with Severe Traumatic Brain Injury

**DOI:** 10.1155/2012/606179

**Published:** 2012-09-26

**Authors:** Ryta E. Rzheutskaya

**Affiliations:** Department of Anesthesiology and Intensive Care, Belarusian State Medical University, Dzerzhinsky Avenue 83, 220116 Minsk, Belarus

## Abstract

*Purpose*. To define specific features of central hemodynamic parameter changes in patients with isolated severe traumatic brain injury (STBI) and in patients with clinically established brain death and to determine the required course of treatment for their correction. *Data and Research Methods*. A close study of central hemodynamic parameters was undertaken. The study involved 13 patients with isolated STBI (group STBI) and 15 patients with isolated STBI and clinically established brain death (group STBI-BD). The parameters of central hemodynamics were researched applying transpulmonary thermodilution. *Results*. In the present study, various types of hemodynamic reaction (normodynamic, hyperdynamic, and hypodynamic) were identified in patients with isolated STBI in an acute period of traumatic disease. Hyperdynamic type of blood circulation was not observed in patients with isolated STBI and clinically established brain death. Detected hemodynamic disorders led to the correction of the ongoing therapy under the control of central hemodynamic parameters. *Conclusions*. Monitoring of parameters of central hemodynamics allows to detect the cause of disorders, to timely carry out the required correction, and to coordinate infusion, inotropic, and vasopressor therapy.

## 1. Introduction

Pathophysiological changes arising after primary brain injury lead to the secondary brain injury [1–3]. Both prehospital and inhospital hypotensions have been shown to have a deleterious influence on outcome from severe traumatic brain injury (STBI) [2, 4–6]. The development of hypotension in patients with STBI can be caused by the reduction of systematic vascular resistance as a result of injury of diencephalic region, the increase of cerebral dislocation signs, and the development of adrenal insufficiency. Another reason for hypotension can be a drop of cardiac output due to the reduction of contractility or hypovolemia, which develops as a result of fluid loss during bleeding, dehydration therapy, diabetes insipidus, and hyperthermia. Hypovolemia initiates the centralization of blood circulation which subsequently brings a number of adverse effects, such as stasis and sludge of erythrocytes in capillaries, ischemia of organs and tissues, tissue edema, and multiple organ failure. Neurogenic Stunned Myocardium (NSM) is still another reason for hypotension, but it has rarely been reported in association with STBI [7]. The main purpose of the ongoing therapy is to prevent and correct hypotension (systolic blood pressure (SBP) < 90 mmHg) [4, 8, 9] and to maintain the target figures of cerebral perfusion pressure (CPP) in the range of 50–70 mmHg [8, 10, 11].

Traumatic brain injury is one of the main causes of brain death in the intensive care units. One of the key problems that arise in the majority of donors with brain damage is acute cardiovascular insufficiency, where hypovolemia plays a special role [12]. The more evident hypovolemia is, the higher the concentration of interleukin-6 and the lower the survival rate of transplant are [13, 14]. One more reason for hypotension may be myocardial dysfunction which is observed in 40% of brain death cases [15, 16]. 

A clear pathophysiological conception of hemodynamic disorders in patients with STBI and in patients with established brain death is an important premise for the rational plan of infusion and inotropic/vasopressor therapy. 

The purpose of the present research was to define specific features of central hemodynamic parameter changes in patients with isolated severe traumatic brain injury and in patients with isolated severe traumatic brain injury and clinically established brain death and to determine the required course of treatment for their correction.

## 2. Materials and Methods

 Meeting the goals of the National Vietnamese Scientific Research: Providing Kidney and Liver Transplantation from Brain-Dead Donors Program, a close study of central hemodynamic parameters was undertaken at the Intensive Therapy Division of Viet Duc University Hospital (Hanoi, Vietnam) from October 2009 until June 2010.

The study involved 13 patients with isolated STBI (group STBI) and 15 patients with isolated STBI and clinically established brain death (group STBI-BD). The level of consciousness was assessed using Glasgow Coma Scale Score (GCS).

Inclusion criteria for group STBI were (1) isolated STBI; (2) GCS 4–7 points on admission to the intensive care unit (ICU); (3) age 18 and older; (4) acute period of STBI (not later than 48 h from the moment of getting trauma. Patients were not always brought to the ICU right after the operation; they could have been held in the emergency recovery room for a period lasting from several hours to 2 days, lacking necessary facilities for central hemodynamic monitoring); (5) absence of concomitant diseases.

Inclusion criteria for group STBI-BD were (1) isolated STBI as a reason of brain death; (2) GCS 3 points; (3) age 18 and older; (4) established brain death (clinical observation, proved using EEG, cerebral angiography); (5) absence of concomitant diseases. The main reasons for STBI were road traffic accidents (a motorbike accident) and falls from height.

The parameters of central hemodynamics were researched applying PiCCO technology using PiCCO2 monitor produced by PULSION Medical Systems (Germany), as well as Philips IntelliVue MP30 patient monitor with the hemodynamic PiCCO-Technology Module M3012A#C10 produced by Philips Medical Systems. All patients had a central venous catheter inserted in the subclavian vein and a 4F 16 cm femoral arterial catheter used for transpulmonary thermodilution (PulsioCath PV2014L16; Pulsion Medical Systems, Munich, Germany). PiCCO technology is based on a combination of two methods: transpulmonary thermodilution and arterial pulse contour analysis [17]. The combination provides continuous measurement of myocardial contractility and volumetric preload, control of afterload, monitoring of cardiac response to volume loading, and interstitial fluid balance in the lungs [18–20]. Triplicate central venous injections of 15 mL ice-cold saline (<8°C) were performed. The module showed the following parameters: mean arterial blood pressure (MAP, mmHg), cardiac index (CI, normal value 3.0–5.0 L/min/m^2^), stroke volume index (SVI, normal value 40–60 mL/m^2^), systemic vascular resistance index (SVRI, normal value 1200–2000 dyn∗s∗cm^−5^∗m^2^), global end-diastolic volume index (GEDI, normal value 680–800 mL/m^2^), stroke volume variation (SVV, normal value ≤ 10%), global ejection fraction (GEF, normal value 25–35%), cardiac function index (CFI, normal value 4.5–6.5 l/min). As an additional parameter extravascular lung water index (ELWI, normal value 3.0–7.0 mL/kg) was also determined. ELWI allows to detect the content of fluid in the pulmonary interstitium. In case of increasing ELWI the type of edema can be defined using pulmonary vascular permeability index (PVPI, normal value 1.0–3.0) [21]. The number of thermodilution measurements for the calibration of continuous CI measurement was from 3 to 7 times a day, subject to the condition of hemodynamics. central venous pressure (CVP, normal value 2–10 mmHg) was monitored before each thermodilution measurement.

Group STBI patients were monitored in an acute period of traumatic disease from the first day in the ICU for 7 days in the light of the ongoing therapy (infusion therapy, vasopressor support). Group STBI-BD patients' central hemodynamic parameters were monitored for 1–3 days from the onset of brain death establishment in the light of the ongoing therapy.

Intracranial pressure monitoring was provided using “Integra NeuroSciences Camino MPM1” monitor in 4 patients of group STBI. It was performed simultaneously with the central hemodynamic parameters monitoring. A Camino catheter was placed into the parenchyma. The monitoring of the intracranial pressure (ICP) was conducted in real time. The cerebral perfusion pressure (CPP) was calculated according to the following formula: CPP = MAP − ICP.

The statistical processing of the data was carried out using specialized software (MS Excel, Statistica 6.0, Biostatistics for Windows, version 4.03). Student's *t*-test was used to estimate the significance of intergroup differences. Single factor analysis of variance followed by Student's *t*-test with Bonferroni correction was used to estimate the significance of intragroup differences. The results are presented in the following format: *M* ± *σ* (mean ± standard  deviation). Spearman's Rank Correlation Method was used to assess the degree of correlation between parameters. A *P* value of 0.05 or less was considered statistically significant.

## 3. Results

The demographic and clinical characteristics of the patients of group STBI are presented in Table 1. The brain CT scan revealed compression of the brain by acute intracranial hematomas (epidural and subdural), associated with severe contusion in 11 (84.6%) patients. They required a surgery. 2 patients had severe brain contusion with accompanying traumatic subarachnoid hemorrhage (SAH). They were not operated. The condition of the patients was assessed critical. The patients entered the ICU either right after operation, or after staying in the emergency recovery room. The patients who did not need the operation entered the ICU right away. 7 patients (53.8%) required hemotransfusion on the first day. Dehydration therapy using Mannitol 20% (0.25–1 g/kg) was given by indication (according to clinical signs of intracranial hypertension or results of monitoring of ICP). In condition of sedation (fentanyl 0.6–0.9 *μ*g/kg/h and midazolam 0.025–0.035 mg/kg/h) pressure control mechanical ventilation was carried out to all patients. All patients received antibacterial therapy and infusion therapy: on the first day isotonic crystalloid and colloid solutions were infused at the average rate of 98.2 ± 43 mL/kg/day. To reach the target value of SBP higher than 90 mmHg and to maintain the value of CPP within the range of 50–70 mmHg, in 8 patients (62%) vasopressor support with norepinephrine infusions at the average rate of 0.12 ± 0.04 *μ*g/kg/min was used prior to the monitoring of the parameters of central hemodynamics (Table 1).

**Figure 1:**
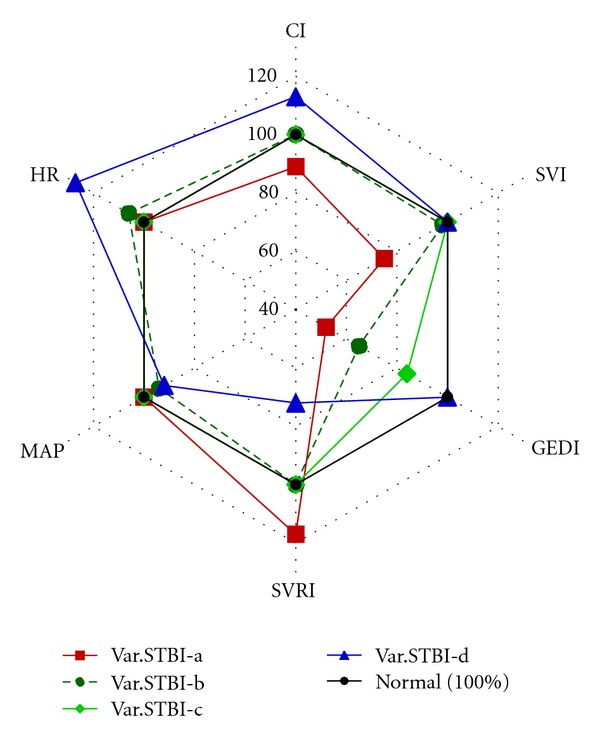
Variants of hemodynamic reaction in the patients of group STBI on the first day. Note. Hemodynamic parameters are presented with percents from the normal value.

Based on the assessment of central hemodynamic parameters obtained on the first day, 4 variants were detected depending on the type of hemodynamic response in patients of group STBI (Figure 1).

Variant STBI-a patients (4 (30.8%) patients) had a hypodynamic type of blood circulation (CI 2.68 ± 0.48 L/min/m^2^) with a high systemic vascular resistance. They had apparent hypovolemia with significant decrease in preload—GEDI ranged from 247 to 473 mL/m^2^ (mean, 353 ± 71.7 mL/m^2^). Their SVI decreased to 30.2 ± 5.1 mL/m^2^. SVV, which assesses cardiac responsiveness to volume loading, exceeded its normal value of less than or equal to 10% and reached 18.5 ± 3%. No apparent tachycardia was observed (HR 91.9 ± 9 beats/min). MAP amounted to 95.8 ± 6 mmHg. Hemodynamic measurements taken after volume loading with Voluven (6% hydroxyethyl starch) 500 mL showed a significant rise of SVI by 10% (*P* < 0.05) in patient no. 4. Moreover, SVI increased by more than 15% in comparison to the initial index in other 3 patients (75%) of this hemodynamic variant. There was an increase of CI, GEDI, GEF, CFI, and decrease of SVV (*P* < 0.05). So, the infusion therapy using crystalloids and colloids (at the average rate of 110 mL/kg/day) was extended. The doses of norepinephrine under SVRI monitoring were reduced to 0.02 *μ*g/kg/min in patient no. 1 and to 0.2 *μ*g/kg/min in patient no. 4. As the result of the ongoing therapy in patients no. 1, 2, 3 by the end of the 1st day of the treatment held under the control of the central hemodynamic parameters monitoring the hypodynamic type of blood circulation with a high systemic vascular resistance changed into the normodynamic one with normal systemic vascular resistance (Figure 3). The condition was preserved on the following day. In patient No. 1 titration of norepinephrine under SVRI monitoring at 0.02 *μ*g/kg/min was run for three days and got cancelled afterwards. In patient No. 2 by the end of the 2nd day a decline of CI with bradycardia and some drop of SVRI were detected. As a result, titration of adrenaline at 0.1 *μ*g/kg/min was used for 5 days. In patient No. 3 at the end of the 2nd day normodynamic type of blood circulation with normal systemic vascular resistance transformed into hyperdynamic type of blood circulation with a low systemic vascular resistance. In the run of the norepinephrine titration at 0.13 *μ*g/kg/min lasting for several hours, SVRI and CI were normalized. The dose of norepinephrine was reduced to 0.1 *μ*g/kg/min on the 3rd day, after which it was cancelled. Starting from the 2nd day the infusion volume reached on average 40 mL/kg/day in these three patients.

 Patient No. 4 had hypotension from the moment of entering the hospital and received a combination of adrenaline at 0.17 *μ*g/kg/min and norepinephrine at 0.27 *μ*g/kg/min (Table 1) combined with the ongoing infusion therapy. By the end of the 1st day of trauma, diabetes insipidus developed. Central hemodynamics parameter monitoring, started at the beginning of the 2nd day of trauma, revealed the above-mentioned type of hemodynamics characterized by apparent hypovolemia with significant decrease in preload (the lowest GEDI 247 mL/m^2^). Despite the extension of volume loading and the decrease in the dose of norepinephrine, hypodynamic type of blood circulation with a high systemic vascular resistance was preserved in the patient (Figure 4). On the 2nd day of the monitoring, at the background of hypodynamic type of blood circulation a sharp decline of SVRI was registered in spite of the increase in the dosage of adrenaline and norepinephrine, leading to the subsequent circulatory arrest.

Variant STBI-b patients (3 (23.1%) patients) had normodynamic type of blood circulation (CI 3.7 ± 0.8 L/min/m^2^) with normal systemic vascular resistance. Some decrease in preload—GEDI ranged from 368 to 587 mL/m^2^ (mean, 494 ± 56.6 mL/m^2^)—and a slight decrease of SVI to the level of 39.3 ± 6.86 mL/m^2^ were observed. SVV exceeded its normal value (20.3 ± 9.2%). No apparent tachycardia was observed (HR 95.6 ± 20.1 beats/min). MAP amounted to 84.8 ± 8.8 mmHg. Hemodynamic measurements taken after a volume loading with Voluven 500 mL showed a significant rise of SVI by 11–27% (*P* < 0.05). In 2 (67%) out of 3 patients SVI increased by more than 15% from the initial value. An increase of CI, GEDI, GEF, CFI and decrease of SVV (*P* < 0.05) were detected. On the first day, under the control of hemodynamic monitoring and in accordance with the response to volume loading, isotonic crystalloid and colloid solutions were infused at the average rate of 70 mL/kg/day. During the 1st day of the monitoring hypovolemia was corrected in all patients (Figure 5). Starting the 2nd day, the infusion volume in these patients was given with an average rate of 35–40 mL/kg/day. 

 Patient No. 5 was provided with invasive monitoring of ICP. In the run of the monitoring, intracranial hypertension was registered. Therefore, the patient was receiving mannitol from day 1 to day 7. All patients of variant STBI-b received norepinephrine for 10–15 days starting from the first day. On the 4th day, a decline of SVRI was detected in patient No. 7. It called for the increase of the doses of norepinephrine.

Variant STBI-c patients (4 (30.8%) patients) had normodynamic type of blood circulation (CI 4.08 ± 0.6 L/min/m^2^), with normal SVRI. GEDI rose from 378 to 795 mL/m^2^ (mean, 573 ± 85 mL/m^2^). SVI and SVV were within normal values. No tachycardia was observed (HR 77.2 ± 10.4 beats/min). MAP increased to 90.5 ± 11.2 mmHg. Hemodynamic measurements taken after volume loading with Voluven 500 mL showed a significant rise of SVI, by 11–27% (*P* < 0.05). SVI increased by more than 15% in comparison to the initial index in 3 (75%) of a total of 4 patients. An increase of CI, GEDI, GEF, and CFI (*P* < 0.05) was detected. On the first day, under the control of hemodynamic monitoring in accordance with the response to volume loading isotonic crystalloid and colloid solutions were infused at the average rate of 50 mL/kg/day. During the 1st day of the monitoring hypovolemia was eliminated in all patients. Starting the 2nd day, the infusion volume in these patients was given with an average rate of 30–35 mL/kg/day.

To patients No. 8 and No. 9 invasive monitoring of ICP was provided. In patient No. 9 intracranial hypertension was registered, so the patient received mannitol on the 4th and the 5th days. Patient No. 10 received norepinephrine only during the operation. On the 4th day, a decline of SVRI was detected in patient No. 9. That required an increase of the norepinephrine doses, lasting for 14 days. Patient No. 11 did not need any norepinephrine. Patient No. 8 received norepinephrine starting from the first day. On the 3rd day in order to normalize SVRI the dose was increased. Moreover, on the 8th day a combination of adrenaline at 0.12 *μ*g/kg/min was used.

Variant STBI-d patients ((15.4%) 2 patients) had a hyperdynamic type of blood circulation (CI 5.65 ± 0.8 L/min/m^2^) with a low systemic vascular resistance. They had normovolemia with GEDI 705.1 ± 84.5 mL/m^2^ (GEDI amounted from 584 to 880 mL/m^2^). SVI and SVV were within normal values. Tachycardia (HR 115 ± 18 beats/min) was observed. MAP amounted to 83.1 ± 2.4 mmHg. Hemodynamic measurements taken after a volume loading with Voluven 500 mL showed a significant rise of SVI by 18% in comparison with the initial index in patient No. 12. An increase of CI, GEDI, GEF, and CFI (*P* < 0.05) was detected. On the first day, under the control of hemodynamic monitoring in accordance with the response to volume loading isotonic crystalloid and colloid solutions were infused at the average a rate of 35 mL/kg/day. 

Taking into consideration the low level of SVRI along with norepinephrine usage by patient No. 12, his dose was raised to 0.17 *μ*g/kg/min and the titration was kept on for the following 7 days. For the correction of the low SVRI in patient No. 13 the titration of norepinephrine at the dose of 0.07 *μ*g/kg/min was started and lasted for the following three days.

ELWI rose in both patients of group 4 without changes in arterial blood gas analyses and in roentgenologic results and reached 10 ± 1.8 mL/kg. It required infusion load limitation and the stimulation of diuresis (lasix) that allowed to normalize ELVI. As a result of the ongoing therapy by the end of the 1st day of monitoring the normodynamic type of blood circulation with normal SVRI was registered in the patients (Figure 6).

To patient No. 13 invasive monitoring of ICP was provided. Intracranial hypertension was registered, so the patient received mannitol on the 4th and 5th days. 

The demographic and clinical characteristics of the patients of group STBI-BD are presented in Table 2. 9 (60%) patients suffered from compression of the brain by acute intracranial hematomas (epidural and subdural), associated with severe contusion. 8 (53.3%) patients required surgery before the brain death establishment. 3 patients had severe brain contusion. 3 patients had severe brain contusion with accompanying traumatic SAH. Infusion therapy: isotonic crystalloid and colloid solutions were infused at the average rate of 69.3 ± 16 mL/kg/day on the first day of brain death establishment. 8 patients (53.3%) required hemotransfusion on the first day. To reach the target of MAP higher than 70 mmHg vasopressor support was carried out in all patients. In 9 (60%) patients hypernatremia was observed, and in 11 patients (73.3%) diabetes insipidus was detected (Table 2). 

**Figure 2:**
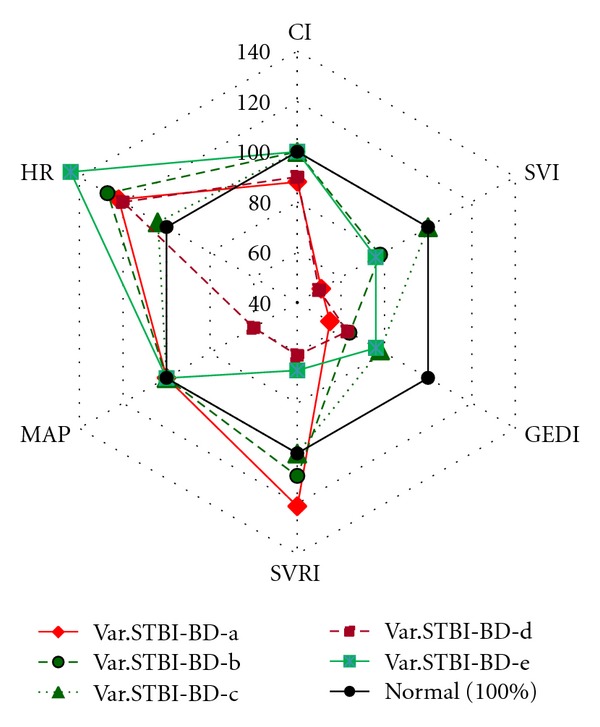
Variants of hemodynamic reaction in the patients of group STBI-BD on the first day. Note. Hemodynamic parameters are presented with percents from the normal value.

**Figure 3:**
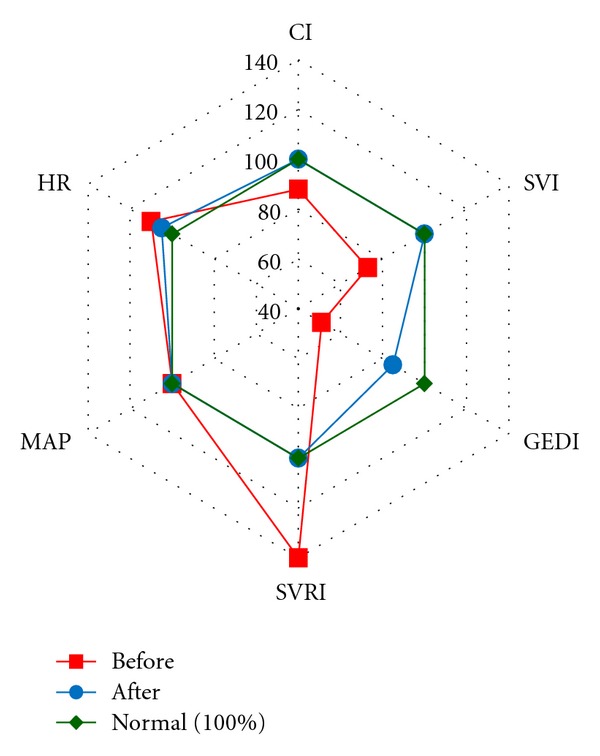
Variant STBI-a of hemodynamic reaction on the first day of monitoring before and after correction. Note. Hemodynamic parameters are presented with percents from the normal value.

**Figure 4:**
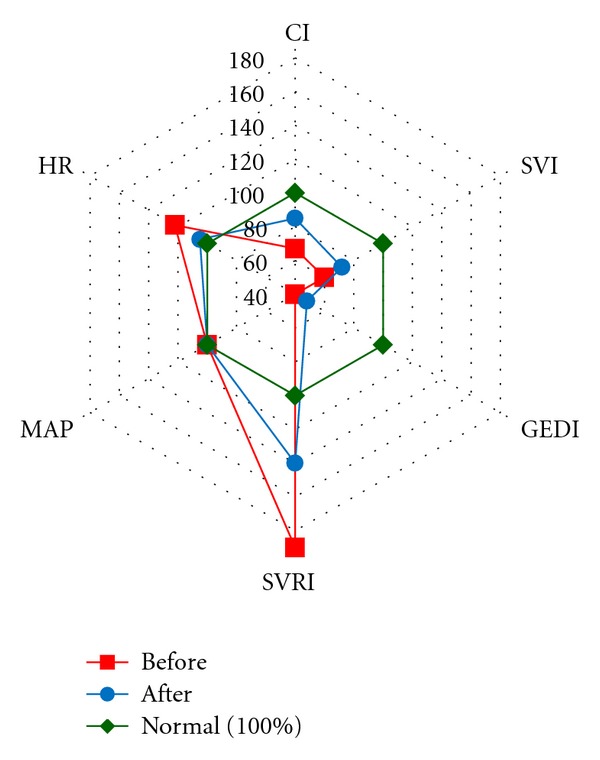
Variant STBI-a of hemodynamic reaction—patient no. 4 on the first day of monitoring before and after correction. Note. Hemodynamic parameters are presented with percents from the normal value.

**Figure 5:**
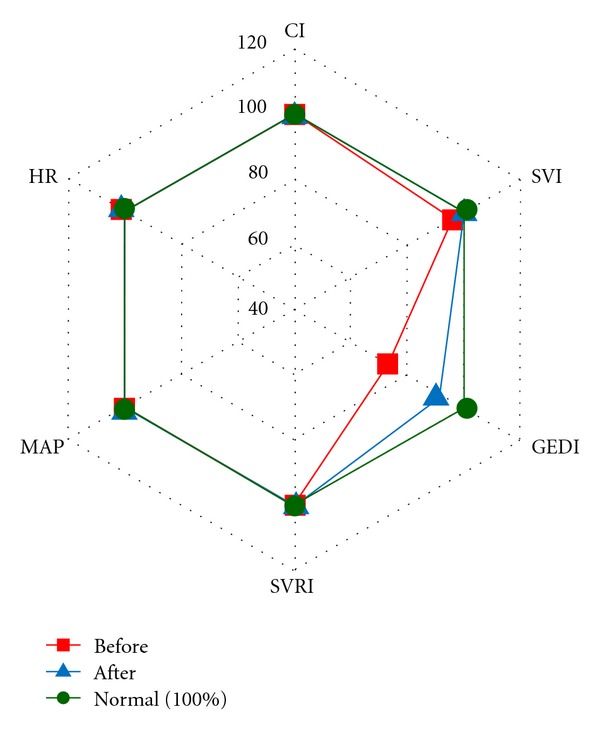
Variant STBI-b of hemodynamic reaction before (the first measment) and after correction in the patients of group STBI on the first day. Note. Hemodynamic parameters are presented with percents from the normal value.

**Figure 6:**
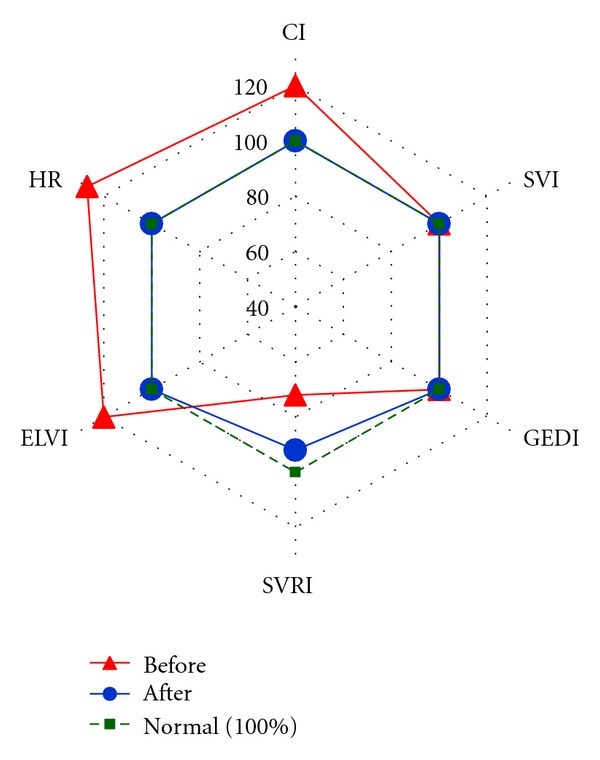
Hyperdynamic type of blood circulation in the patients of group STBI on the first day before and after correction. Note. Hemodynamic parameters are presented with percents from the normal value.

Based on the assessment of central hemodynamic parameters obtained on the first day, 5 variants depending on the type of hemodynamic response were detected in patients of group STBI-BD (Figure 2).

Variant STBI-BD-a patients (6 (40%) patients) had a hypodynamic type of blood circulation (CI 2.65 ± 0.36 L/min/m^2^) with a high systemic vascular resistance. They had apparent hypovolemia with significant decrease in preload—GEDI ranged from 270 to 615 mL/m^2^ (mean, 373.6 ± 81.9 mL/m^2^). Their SVI decreased to 20.5 ± 8.51 mL/m^2^. SVV, which assesses cardiac responsiveness to volume loading, significantly exceeded its normal value of less or equal to 10% and reached 22.85 ± 8.1%. Tachycardia (HR 110 ± 11.4 beats/min) was observed. MAP amounted to 89.6 ± 20.7 mmHg. Hemodynamic measurements taken after volume loading with 500 mL Voluven showed a significant rise of SVI by less than 15% in comparison with its initial index, and SVI ranged from 20 to 43% (*P* > 0.05) in all patients. An increase of CI, GEDI, GEF, CFI and decrease of SVV (*P* < 0.05) were detected.

In patients No. 1 and 5 at the end of the 1st day of monitoring in the light of the ongoing infusion therapy, the dose of norepinephrine was reduced. Hypodynamic type of blood circulation with a high systemic vascular resistance transformed into a normodynamic type of blood circulation with normal systemic vascular resistance, but on the 2nd day hypodynamic type of blood circulation with a low systemic vascular resistance was registered which required the increase of the doses of vasopressors. In spite of the ongoing therapy, in patients No. 2, 3, 4, 6 (Table 2) hypodynamic type of blood circulation was preserved which apparently was connected with polyuria, badly corrected with minirin and disturbed vascular tone central regulation.

Variant STBI-BD-b patients (5 (33.3%) patients) had a normodynamic type of blood circulation (CI 3.4 ± 0.4 L/min/m^2^) with high systemic vascular resistance. Some decrease in preload—GEDI from 270 to 736 mL/m^2^ (mean, 433.6 ± 76.5 mL/m^2^)—and a decrease of SVI to the level of 31.4 ± 5 mL/m^2^ were observed. SVV exceeded its normal value (19 ± 8.3%). Tachycardia (HR 114.8 ± 12.5 beats/min) was observed. MAP amounted to 93.44 ± 12.7 mmHg. Hemodynamic measurements taken after a volume loading with 500 mL Voluven showed a significant rise of SVI by less than 15% in comparison with its initial index, (*P* < 0.05) in 4 (80%) patients of 5 patients. There was an increase of CI, GEDI, GEF, CFI and decrease of SVV (*P* < 0.05). In patients No. 8 and 9 in the light of the ongoing therapy (Table 2) normodynamic type of blood circulation with high systemic vascular resistance was preserved. In patients No. 7, 10, 11 on the 2nd day of the monitoring hypodynamic type of blood circulation with a high systemic vascular resistance in light of increase of vasopressors doses was registered. 

Variant STBI-BD-c patients (2 (13.3%) patients) had a normodynamic type of blood circulation (CI 4.07 ± 0.6 L/min/m^2^) with normal systemic vascular resistance. GEDI decreased and reached the level of 530 ± 68.5 mL/m^2^ (ranged from 404 to 652 mL/m^2^). SVI (44.25 ± 4.8 mL/m^2^) and SVV (9.75 ± 3%) were within normal values. No apparent tachycardia was observed (HR 93.9 ± 8.2 beats/min). MAP amounted to 95.3 ± 15.9 mmHg. In patient No. 13 at the background of the ongoing therapy (Table 2) and decreased norepinephrine doses normodynamic type of blood circulation with normal systemic vascular resistance was preserved during the following days. In patient No. 12 on the 2nd day of the monitoring hypodynamic type of blood circulation with a normal systemic vascular resistance was registered at the background of the titration of norepinephrine.

Variant STBI-BD-d patients (1 (6.67%) patient) had hypodynamic type of blood circulation (CI 2.7 ± 0.2 L/min/m^2^) with low systemic vascular resistance despite high doses of vasopressors. He had apparent hypovolemia with GEDI 427 mL/m^2^. His SVI was very low. Tachycardia (HR 108.5 ± 2 beats/min) was observed. This type of blood circulation was registered 30 minutes before the development of circulation arrest.

Variant STBI-BD-e patients (1 (6.67%) patient) had normodynamic type of blood circulation (CI 3.95 ± 0.8 L/min/m^2^) with low systemic vascular resistance. GEDI decreased to 513.3 ± 26.7 mL/m^2^. SVI was low (30.5 ± 1.7 mL/m^2^). Apparent tachycardia (HR 130 ± 14 beats/min) was observed. MAP amounted to 90.3 ± 12.7 mmHg. Despite the increase in the dose of norepinephrine low SVRI was preserved. Hemodynamic measurements taken in patients with variants STBI-BD-c, STBI-BD-d, STBI-BD-e after a volume loading with Voluven 500 mL did not show any significant rise of SVI in comparison to its initial value.

## 4. Discussion

So, in 31% of patients of group STBI and in 47% of patients of group STBI-BD the hypodynamic type of blood circulation was detected, and a significant hypovolemia was highlighted (GEDI amounted to 52–55% from the norm, SVV was increased by more than 10%) that led to the drop of SVI (compounded 51–75% from the normal value) and also to the drop of cardiac output. Global ejection fraction (GEF) that characterizes a contractile myocardium function was within normal values in all patients of group STBI and STBI-BD that indicated the absence of cardiac failure.

Apparently the development of hypodynamic type of blood circulation points at the failure of compensatory mechanisms of the blood circulation. It may develop as a result of an absolute hypovolemia which is associated with a significant loss of circulatory blood volume (bleeding, dehydratation therapy, diabetes insipidus, hyperthermia), and also due to a reliable hypovolemia which is associated with the increase of volume of bloodstream as a result of SVR decrease which was due to the disturbed vascular tone central regulation. If the development of hypodynamic type of blood circulation is mainly connected with the loss of circulatory blood volume, its compensation leads to the change of this type of blood circulation into normodynamic one. If there is a combination of an absolute and relative hypovolemia the implementation of vasopressors with infusion therapy is required. If it is impossible to transform the hypodynamic type of blood circulation into the normodynamic one for several hours, it points at a significant (irreversible) damage of central vasoconstrictive mechanisms. It was not possible to manage the transformation of the hypodynamic type of blood circulation into the normodynamic type in one (lethal outcome) of 4 patients of group STBI and in 4 patients of 6 patients of group STBI-BD. So, the hypodynamic type of blood circulation is unfavourable in a prognostic way. These findings are consistent with other studies [22, 23] demonstrating that survivors after trauma had higher CI than nonsurvivors. Patients with head injuries who subsequently became brain dead initially had low CI with poor tissue perfusion beginning shortly after emergency department admission [24].

Normodynamic type of blood circulation was observed in 54% of patients of group STBI and in 53% of patients of group STBI-BD who suffered from hypovolemia (GEDI amounted to 64–84% from normal value) which is less expressed in comparison with the patients with hypodynamic type of blood circulation. SVI was normal or slightly decreased. Normal cardiac output was provided by tachycardia with decreased SVI. For the correction of absolute hypovolemia in patients with the hypodynamic type of blood circulation a larger volume of infusion therapy was required than in patients with normodynamic one.

Normodynamic type of blood circulation in the majority of patients of group STBI-BD transformed into hypodynamic type. Normodynamic type of blood circulation in 1 of the patients of group STBI transformed into hyperdynamic type on the following day. The rest of the patients of group STBI preserved the normodynamic type.

Hyperdynamic type of blood circulation was not observed in group STBI-BD patients (Figure 2) that apparently was connected with the hemodynamic monitoring carried out in the background of the ongoing vasopressor support. Nevertheless, one study [24] shows the hyperdynamic state in brain-dead patients.

To make a conclusion, normodynamic and hyperdynamic types of blood circulation are more favourable than hypodynamic type.

In 3 patients of 4 who were provided with ICP monitoring intracranial hypertension was characterized by the decrease of SVRI. The correction of doses of norepinephrine under SVRI monitoring was required to patients with a systemic vascular resistance deficit (15% patients) and with excess of systemic vascular resistance (31% patients) of group STBI.

All the patients of group STBI-BD were provided with vasopressor support to maintain the target parameters of MAP (70–90 mmHg). 12% of patients with a low systemic vascular resistance and 73% of patients with a high systemic vascular resistance required increase or decrease of norepinephrine under SVRI monitoring. Norepinephrine use in this category of patients was a reasonable choice. When combination of hypotension and bradycardia was observed, adrenaline was the choice. Apparently all patients of group STBI-BD had a combination of absolute and reliable hypovolemia that is why they needed vasoconstrictive drugs given in larger doses than to patients of group STBI. At the beginning period of brain death the increase of vasopressors doses leads to the sharp rise of SVRI. But at later periods of brain death SVRI does not respond so much to the increase of vasopressors doses (Variants STBI-BD-d, STBI-BD-e). These findings are consistent with other studies [25, 26] demonstrating that low systemic and pulmonary vascular resistances have been documented in the majority (75%) of brain dead subjects. In the late or end stage of brain death, hemodynamic deterioration and collapse led rapidly to arrest [24].

Our results demonstrated that hypovolemia is often detected in patients with isolated STBI in an acute period of traumatic disease and in brain dead donors. These findings are consistent with other studies [13, 27]. The hypovolemic state is difficult to assess without monitoring of central hemodynamic parameters.

One of the methods of preload determination which is commonly used nowadays is the measurement of CVP. During the research process normal values of central venous pressure (CVP, mmHg) were observed in all patients. Correlation analysis revealed no significant correlation between CVP and GEDI, a preload parameter. No significant correlation was revealed between CVP and ELWI either. These results are in agreement with other studies [28–31] and confirm the limited value of CVP both as an indicator of cardiac preload and as a predictor of fluid responsiveness. Therefore, monitoring of CVP cannot always determine adequate hemodynamic status of a patient (CVP can be within its normal values with normovolemia and hypovolemia). In the studies by Michard et al. [18, 32, 33], it was demonstrated that global end-diastolic volume (GEDV) but not CVP behaves as an indicator of cardiac preload.

It should be mentioned that not all cases with decreased GEDI SVV were increased. It may be related to the preserved patient initiation of the ventilator. Both GEDI and SVV may help in decision-making process concerning volume loading [28, 34, 35]. In cases of SVV limitation (arrhythmias, spontaneous breathing) GEDI as an indicator of cardiac preload can be applied [29, 30]. As it was shown in the studies of Michard at al., the lower is the preinfusion GEDI, the more marked are the hemodynamic effects of volume loading [18].

Positive response to volume loading which is characterized by the increase of SVI from its initial index by more than 15% was detected in 9 (69%) patients of 13 in group STBI and in 10 (66%) patients of 15 in group STBI-BD. SVI increased from its normal value by more than 10% in 11 (85%) patients of group STBI. So, in patients with detected hypovolemia and positive response to volume loading a pathogenetically based hemodynamic correction was carried out by the extension of infusion therapy. 

Transpulmonary thermodilution enables the identification of patients with pulmonary edema (increased EVLW) as well as the quantification of pulmonary edema and its response to the ongoing treatment (e.g., fluid restriction/depletion) [21, 36, 37]. In addition, the assessment of pulmonary vascular permeability (PVPI) provides a better understanding of the pathophysiological mechanisms of hypoxemia.

Thus, the goal of management for hemodynamic status of the patients with STBI is to avoid hypovolemia by the means of careful fluid management, maintenance of blood pressure for reducing the risk of cerebral ischemia. The goals of management for the donor's hemodynamic status are to achieve normovolemia by volume expansion, maintenance of blood pressure, and optimization of cardiac output so as to reach perfusion pressure and blood flow gradients that promote organ function with the least support of vasoactive drugs [26, 38]. Hemodynamic management requires continuous invasive monitoring to ensure that cardiac preload, afterload, and contractility are optimal [39]. Infusion therapy based on the estimation of routine hemodynamic parameters (blood pressure, heart rate, central venous pressure, daily fluid balance) could not prevent hypovolemia in the examinees and caused a high rate of sympathomimetic use in uncorrected volemic states [27]. Despite a limited scope of observations, our results confirmed the reasonability of hemodynamic monitoring which allows to determine the cause, carry out a timely correction of the observed disorders, and decrease the risk of complications associated with hypotension in patients with STBI and in patients with clinically established brain death. Further studies with more patients will help to reveal new features and regularities of central hemodynamic parameter changes in patients with STBI and to define the required measures for their correction.

## 5. Conclusion

 In the present study, various types of hemodynamic reaction in patients with STBI were identified: normodynamic, hyperdynamic, and hypodynamic. Hyperdynamic type of blood circulation was not observed in patients with STBI and clinically established brain death. Monitoring of parameters of central hemodynamics (CI, SVI, SVRI, GEDI, SVV, GEF, CFI) allows carrying out pathogenetically based infusion, inotropic, vasopressor therapy in patients with STBI and in patients with clinically established brain death.

## Figures and Tables

**Table 1:** Characteristics of patients with STBI (group STBI).

Patients (variants)	Gender, age (years)	GCS, points	Operation	Hemotransfusion	Use of Mannitol	CVP, mmHg	Norepinephrine *μ*g/kg/min	Adrenaline *μ*g/kg/min	Rate of infusion mL/kg/day	Urine output mL/kg/h	Length of stay in the ICU	Outcome
1 (STBI-a)	M, 46	6	+	+	+	5	0.12	—	114	1.6	20	Favorable
2 (STBI-a)	M, 20	6	+	+	+	10	—	—	135	2.8	8	Favorable
3 (STBI-a)	M, 21	7	+	+	−	4	—	—	82	1.5	11	Favorable
4 (STBI-a)	F, 43	6	+	+	−	10	0.27	0.17	116	8.6	3	Death
5 (STBI-b)	M, 39	5	−	−	+	4	0.1	—	48	1.4	37	Favorable
6 (STBI-b)	M, 25	4	+	−	+	5	0.13	—	122	2.9	12	Favorable
7 (STBI-b)	M, 45	6	−	−	+	5	0.12	—	49	3.0	19	Favorable
8 (STBI-c)	M, 60	5	+	−	−	6	0.2	—	50	2.4	14	Death
9 (STBI-c)	M, 49	6	+	+	+	2	0.12	—	35	1.2	21	Favorable
10 (STBI-c)	M, 38	4	+	+	−	10	—	—	54	2.1	13	Favorable
11 (STBI-c)	M, 29	6	+	−	−	5	—	—	47	1.9	9	Favorable
12 (STBI-d)	M, 20	6	+	+	−	10	0.13	—	100	2.8	8	Favorable
13 (STBI-d)	M, 72	6	+	+	−	9	—	—	36	1.5	21	Favorable

GCS, CVP, use of mannitol, doses of norepinephrine and adrenaline are presented at the moment of the monitoring beginning,

rate of infusion, urine output, use of mannitol on the first day of the monitoring,

M: male, F: female.

**Table 2:** Characteristics of patients with STBI and clinically established brain death (group STBI-BD).

Patients (variants)	Gender age (years)	Operation	Hemotransfusion	Diabetes insipidus	CVP, mmHg	Norepinephrine *μ*g/kg/min	Adrenaline *μ*g/kg/min	Rate of infusion mL/kg/day	Urine output mL/kg/h	Use of minirin	Day from trauma
1 (STBI-BD-a)	M, 21	+	+	+	9	0.15	—	54	3.9	−	6
2 (STBI-BD-a)	M, 21	+	−	+	10	0.3	0.1	155	13.8	+	2
3 (STBI-BD a)	M, 38	−	−	+	4	0.4	0.2	94	8.5	+	1
4 (STBI-BD-a)	M, 34	+	−	+	9	0.19	—	62	5.5	+	5
5 (STBI-BD-a)	M, 26	+	+	−	8	0.15	—	75	2.0	−	3
6 (STBI-BD-a)	M, 55	+	+	+	10	0.6	0.19	84	6.25	+	4
7 (STBI-BD-b)	F, 36	−	+	+	7	0.3	0.15	87	4.0	+	2
8 (STBI-BD-b)	M, 38	−	−	+	3	0.3	—	82	3.1	+	3
9 (STBI-BD-b)	M, 26	−	−	+	6	0.16	—	87	9.6	+	2
10 (STBI-BD-b)	M, 18	−	−	+	4	0.15	—	96	4.9	+	4
11 (STBI-BD-b)	F, 24	+	−	−	6	—	0.15	55	2.95	−	6
12 (STBI-BD-c)	M, 53	+	+	+	9	0.19	—	54	2.62	+	9
13 (STBI-BD-c)	M, 25	−	+	−	4	0.22	—	45	1.4	−	4
14 (STBI-BD-d)	M, 64	−	−	−	12	0.28	0.22	58	0.2	−	1
15 (STBI-BD-e)	M, 44	+	+	+	5	0.4	—	84	4.86	+	3

Day from getting trauma, CVP, use of Minirin, doses of norepinephrine and adrenaline are presented at the moment of the monitoring beginning,

rate of infusion, urine Output, on the first day of the monitoring,

M: male, F: female.
